# Focal atrophy of the unilateral masticatory muscles caused by pure trigeminal motor neuropathy: case report

**DOI:** 10.1002/ccr3.1495

**Published:** 2018-03-30

**Authors:** Antti Kämppi, Leena Kämppi, Pentti Kemppainen, Mari Kanerva, Jussi Toppila, Mari Auranen

**Affiliations:** ^1^ Department of Oral and Maxillofacial Diseases Helsinki University Hospital Helsinki Finland; ^2^ Clinical Neurosciences, Neurology University of Helsinki and Helsinki University Hospital Helsinki Finland; ^3^ Clinicum Department of Oral Sciences University of Helsinki Helsinki Finland; ^4^ Inflammation Center Division of Infectious Diseases University of Helsinki and Helsinki University Hospital Helsinki Finland; ^5^ Clinical Neurophysiology Medical Imaging Helsinki University Hospital Helsinki Finland; ^6^ Research Programs Unit Molecular Neurology University of Helsinki Helsinki Finland

**Keywords:** Dentistry, neurology, unilateral trigeminal motor neuropathy

## Abstract

Patients with unknown clinical or radiological asymmetry in the face structures combined with atrophy and weakness of the masticatory muscles should be comprehensively examined clinically and with MRI, neurophysiological measurements, and serologically. Malignant lesions or benign idiopathic unilateral trigeminal motor neuropathy should be considered as an etiological explanation for the asymmetry.

## Introduction

Pure unilateral trigeminal motor neuropathy (UTMN) is a very rare medical condition, which is characterized by paralysis of the motor branch of the trigeminal nerve without disturbances of the sensory branches of trigeminal nerve or any other cranial nerves. This condition was first described by Chia LG et al. in 1988. Altogether twelve UTMN case reports (16 patients) have been published during 1988–2017. Etiological factors for UTMN have been considered to be viral infection 1, 2, 3 (5/16), tumor 4, 5 (2/16), trauma 6 (1/16), stroke 7 (1/16), Sjögren's syndrome 8 (1/16), and unknown 1, 9, 10, 11, 12 (6/16).

Typical symptoms of UTMN, before visible asymmetry of the face, are feeling of wasting and weakness of the masticatory muscles. Some patients have also reported chewing problems and deviation of the jaw. Pure trigeminal motor neuropathy can occur in the main motor branch of the nerve or in its distal branches. Typical findings in MRI are replacement of the muscle tissue by fat tissue with no other explanatory factors (i.e., tumors). Clinically, unilateral attrition in dentition alone or with compensatory overgrowth of the mandible may be present in chronic cases.

## Case History and Examination

The patient is a 57‐year‐old highly educated man, ethnical Armenian, who was born in Uzbekistan and lived childhood in Tadzikistan. Before moving to Finland 17 years ago, he has spent time in the Soviet Union and Greece. He is a nonsmoker and no traumas or accidents in face area have been reported. He has gone through several operations including acute gastric ulcer surgery, tonsillectomy, uvulectomy, and septoplasty. He has never detached ticks from his skin or noticed erythema migrans, although he often spends time outdoors and has potentially been exposed to ticks. Patient has been diagnosed with hypercholesterolemia, premature ventricular beats, and benign prostatic hyperplasia and is being treated with bisoprolol and tamsulosin. Left thyroid block was resected due to microcarcinoma and goiter (2008). He has experienced mild cognitive dysfunction leading to a neurological consultation 1 year earlier, but no specific diagnosis was made.

**Figure 1:**
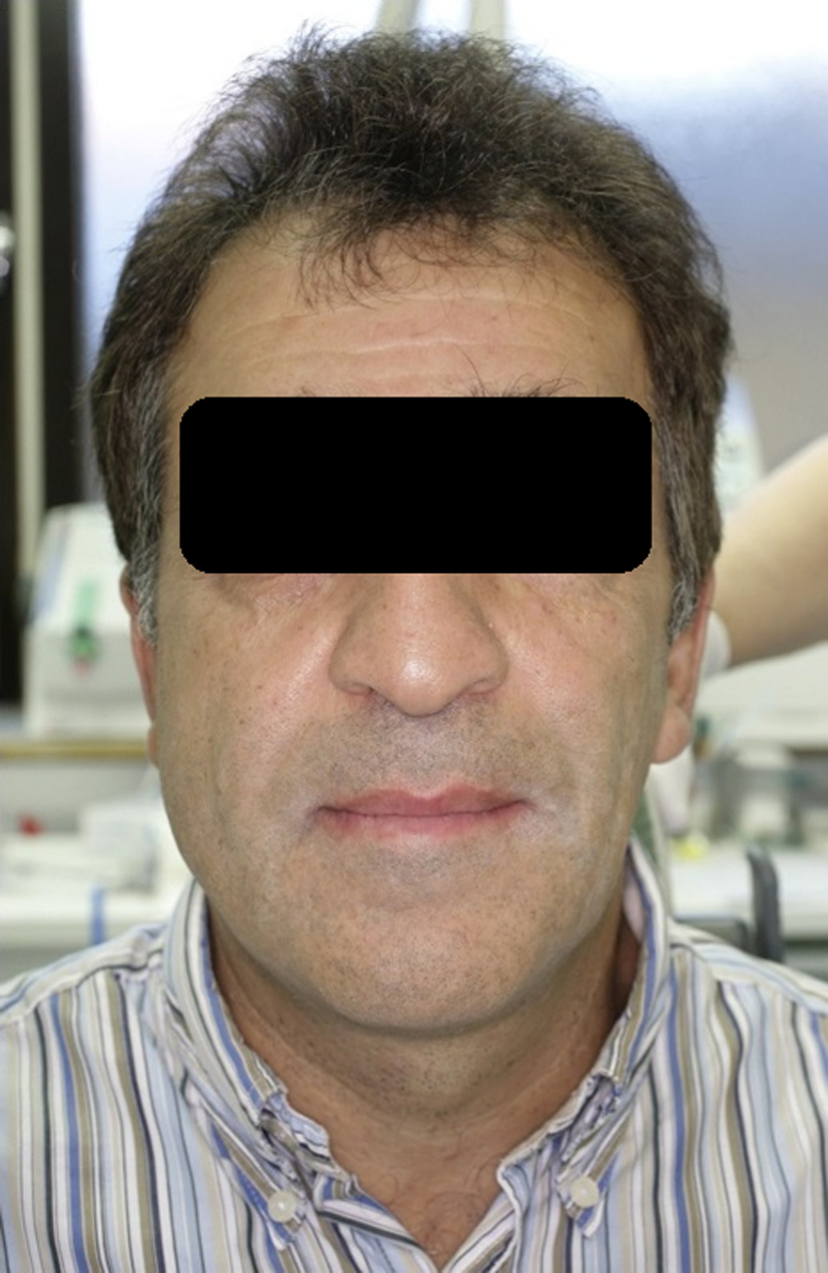
Clear asymmetry is visible between left and right cheeks.

**Figure 2:**
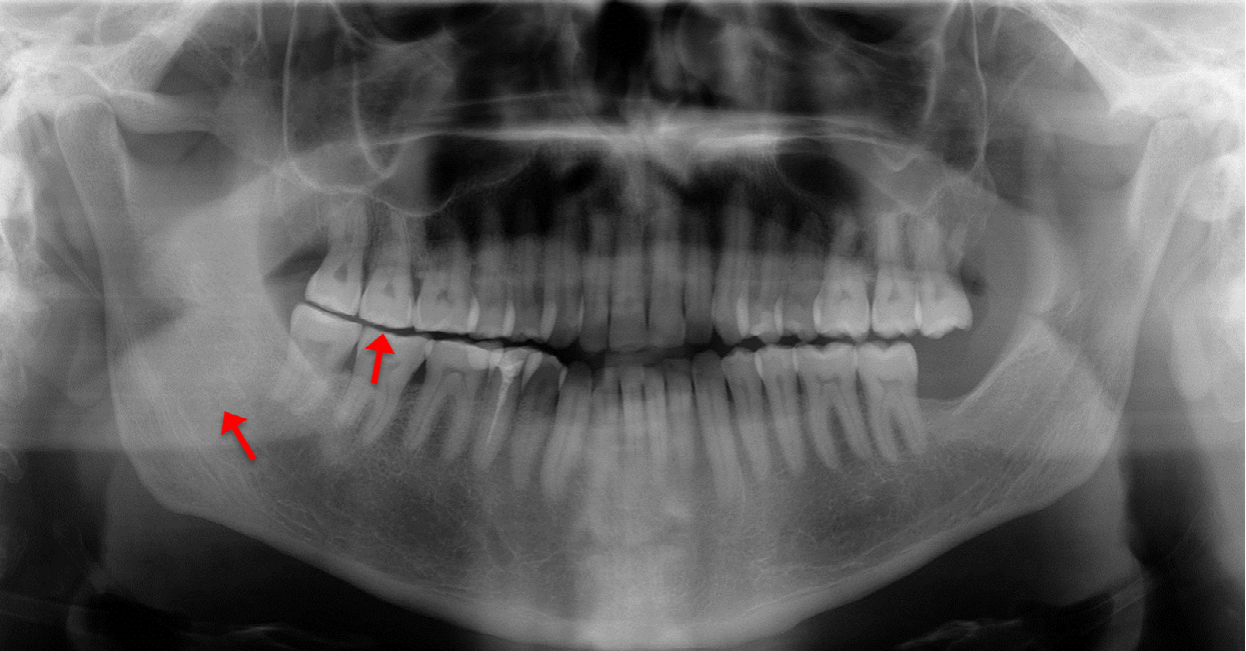
Dental and bone asymmetry between left and right side of the mandible. Corpus area is stronger and attrition visible on the right side (red arrows) compared to the left side.

The patient sought medical assistance for slowly worsening right sided masticatory pain in action, combined with left‐sided masticatory weakness for at least 5 years. Visually patient's face was severely asymmetric (Fig. 1). According to stomatognathic physiological examination, movements and the coordination of mandible were within normal range. In the analysis, free painless jaw opening was 35 mm with no deviations, free lateral movement to right side was 10 mm and to left side 5 mm, protrusion was 10 mm plus horizontal over bite, vertical over bite was −1 mm and horizontal over bite 1 mm, and the patient had no contact to antagonists between teeth 13 and 24. Centric relation in temporomandibular joints was equal to maximum intercuspal position, that is, jaw coordination was stable. Clinically and in orthopantomogram (PTG) (Fig. 2), moderate attrition in right side was detected, whereas left‐side dental cusps were almost intact. Also in the right side, slight mandibular overgrowth was seen.

**Figure 3:**
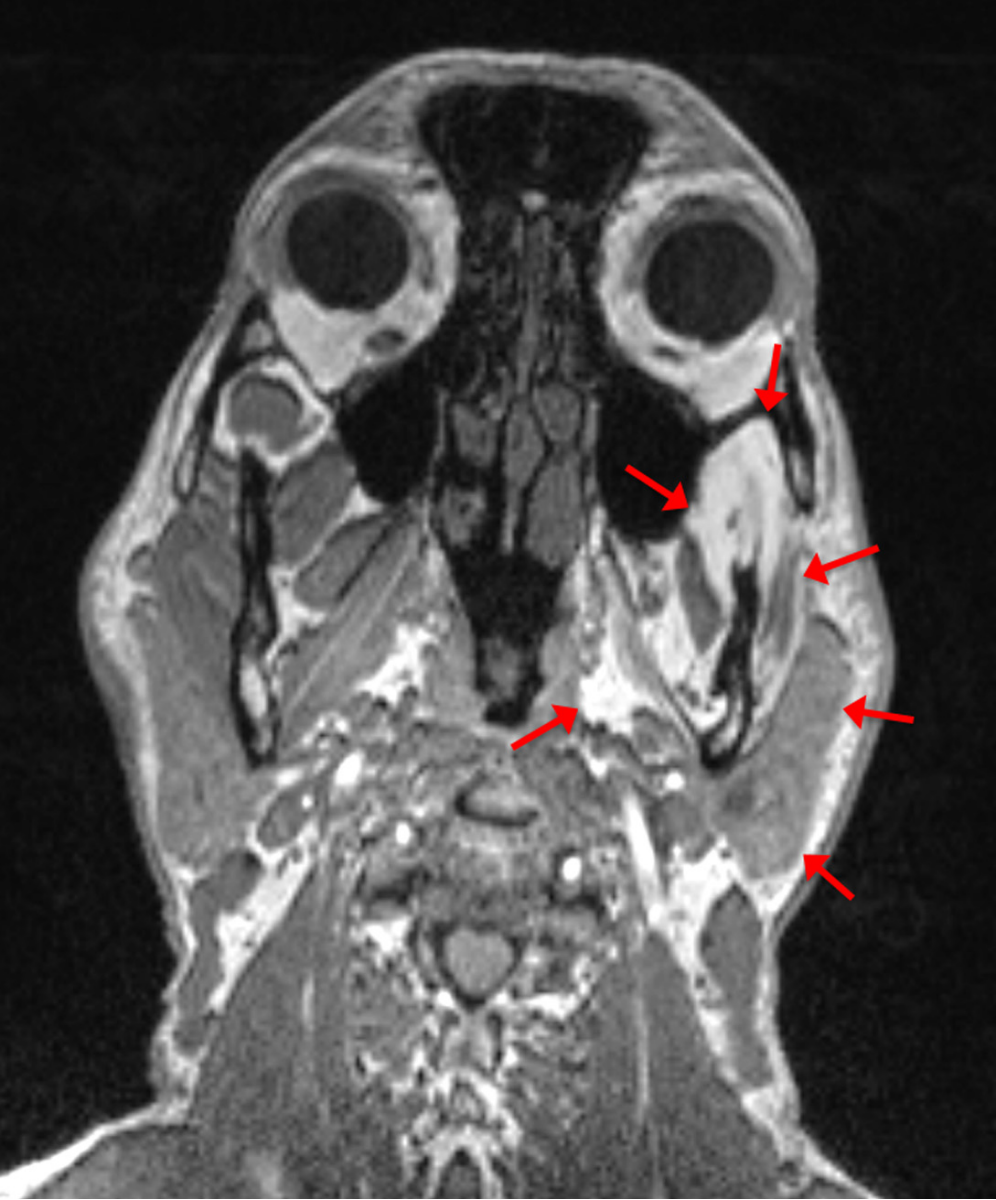
According to MRI scanning, the length of the condylar‐angulus measure asymmetry was 4 mm. Measurements were recorded from axial T1 weighted and coronal T2 weighted images. Muscle tissue replacement with fat tissue and atrophy of masticatory muscles were also seen in MRI scanning (red arrows).

Bite‐related factors were excluded by preparing and using a splint as a generally accepted first‐line treatment to TMJ disorders with muscle pain. The splint, in which bite was prepared physiologically correct, was used for 4 months without any relief of pain or relieving the condition. The patient was then directed to MRI scanning which revealed severe atrophy in all left‐side masticatory muscles (Fig. 3) being widely displaced with fat tissue. There were no clear signs of infection or inflammation around the trigeminal nerve.

In neurological examination, the function and findings of the cranial nerves were intact with the exception of the motor branch of the left trigeminus nerve. No evidence of additional motor nerve involvement or signs of polyneuropathy was detected. The muscle strength, deep tendon reflexes, and sensory testing of limb muscle were normal. In needle myography (EMG), signs consistent with slight to moderate inactive chronic neurogenic defect in all masticatory muscles innervated by left motor branch of the trigeminal nerve (masseter, temporalis, and medial and lateral pterygoid muscles) were present combined with moderate chronic inactive axonal loss in the left trigeminal motor branch. However, no changes were observed on the right side nor in the muscles innervated by left facial nerve (frontalis and orbicular oris muscles) (Table 1). Blink reflex responses R1 and R2 were normally elicited from the left supraorbital, infraorbital, and mental sensory branches of the left trigeminal nerve with normal latencies (Table 2). Needle myography (EMG) was performed with Keypoint Focus 9033A07; Alpine Biomed ApS, and software Keypoint.NET Software v. 2.32.

**Table 1:** EMG findings

Muscle	Innerv	Str	Atr	Spont	UL	Amp	Dur	Poly	Jig	Interpretation
Left medial pterygoid	V	4	2	0	2	0	0	0	0	Moderate chronic neurogenic
Left lateral pterygoid	V	4	n.a.	0	1	0	0	0	0	Slight chronic neurogenic
Left temporal	V	4	2	0	2	0	0	0	0	Moderate chronic neurogenic
Left orbicular oris	VII	5	0	0	0	0	0	0	0	Normal
Left frontalis	VII	5	0	0	0	0	0	0	0	Normal
Left masseter	V	4	2	0	2	1	0	0	0	Moderate chronic neurogenic
Right masseter	V	5	0	0	0	0	0	0	0	Normal

Innerv, Innervation; Str, Strength (0–5); Atr, Atrophy (0–3); Spont, Spontaneous activity (0–3); UL, Motor unit loss (0–3); Amp, Motor unit amplitude (−3 to 3); Dur, Motor unit duration (−3 to 3); Poly, Proportion of polyphasic motor unit potentials (0–3); Jig, Jiggle i.e. motor unit instability (0–3).

**Table 2:** Blink reflex findings

Stim: Left Supraorb. Rec: Orb. oc.	R1‐Lat [ms]	RefLimit	R2‐Lat [ms]	RefLimit	Stim: Left Infraorb Rec: Orb. oc.	R2‐Lat [ms]	RefLimit	Stim: Left Mentalis Rec: Orb. oc.	R2‐Lat [ms]	RefLimit
Left	8.9	<11.0	31.9	<41.0	Left	33.5	<41.0	Left	31.6	<41.0
Right			33.3	<44.0	Right	33.3	<44.0	Right	31.5	<44.0
Difference			−1.35	<7.0	Difference	0.2	<7.0	Difference	0.041	<7.0

All extensive serum laboratory examinations were also normal except that antibodies against Borrelia burgdorferi showing repeatedly highly positive results for Borrelia IgG, (IgG for VlsEAg > 240 AU/mL (reference < 10 VE/mL), but not for IgM (IgM for VlsEAg 15–18 (reference < 18 VE/mL). Also, the immunoblotting for Borrelial IgG antibodies was positive. In cerebrospinal fluid (CSF) analysis, there was no inflammation. Serum chemokine CXCL13 was negative. However, a small amount Borrelia IgG antibody was detected in the CNS fluid, (Li‐BorrAbG 15 wME, reference < 3 wME), but the index for intrathecal Borrelia antibody production as well as Borrelial nucleic acid detection were negative. Although the diagnosis of (neuro)borreliosis could not be firmly established, the patients received 2 g of intravenous ceftriaxone for 3 weeks.

## Discussion

This is the first time that a potential Borrelia burgdorferi infection underlying UTMN symptoms is reported. Previously, the etiology behind UTMN has remained poorly understood. Common denominator for UTMN is weakness of muscles innervated by trigeminal motor branch. In the pure disease entity, sensory branches of trigeminal nerve or other cranial nerves should not be involved. Imaging studies, such as MRI and PTG, and neurophysiological measurements are used to identify idiopathic UTMN and to exclude other causes such as tumors. Other etiological factors were excluded by blood tests. The typical finding in masticatory muscles is replacement of muscles by fat tissue.

Anatomical 13 and clinical studies 14 have shown that masticatory muscles receive bilateral projections from both cortical hemispheres. Therefore, it seems likely that idiopathic UTMN is due to a lesion affecting the subnuclear trigeminal motor tract. This is in line with very few earlier clinical studies 11 indicating that lesions at the level of pons or more distally of trigeminal motor nerve tract result in pure trigeminal nerve motor neuropathy.

In our case, bite‐related etiological factors were excluded using splint for 4 months without any pain relief or change in the occlusion.

According to the literature, the most common factor for UTMN is a postviral infection complication of the upper respiratory track. In our case, there was no history of infections. Also, the brain MRI showed no signs of malignancy or anatomical disturbances of trigeminal motor branch. Highly positive Borrelia burgdorferi antibodies suggested a putative etiological factor in our patient. However, active neuroborreliosis could not be confirmed by the CSF examination. Our patient did not report any previous systemic or other specific symptoms of neuroborreliosis (i.e., meningitis or radicular pain). Nevertheless, considering the long history of symptoms in our patient, at least 5 years, the Borrelia burgdorferi infection underlying UTMN symptoms cannot be definitely ruled out. It could be speculated that an active infection may have healed spontaneously leaving only nerve damage and highly positive serologic findings. Although Borrelia has never been described as an etiology for UTMN before, a newly published report suggests that Borrelia burgdorferi can affect trigeminal nerve and other cranial nerves, although most commonly it affects facial nerve 15.

In summary, our patient went through very comprehensive examinations. According to clinical, serological, radiological, and neurophysiological test results, the diagnosis of left‐sided pure UTMN in proximal part of the trigeminal nerve was established. We conclude that although etiology could not be definitely confirmed, we propose Borrelia burgdorferi as a putative etiological factor in our case.

There was no progression detected in patient's condition during the examination and follow‐up for nearly 1.5 years. Currently, there are no local or international guidelines for treatment of UTMN with stomatognathic involvement in the existing literature. We suggest that a treatment trial of intravenous ceftriaxone 2 g/day for 3 weeks should be initiated for patients with suspected or diagnosed neuroborreliosis.

## Conflict of Interests

None of the authors have any conflicts of interests.

## Authorship

AK: dentist in specialization of prosthodontics and stomatognathic physiology, treating dentist, corresponding author. LK: specialist in Neurology, equally contributed to diagnosing and writing. PK: specialist in dentistry, professor of prosthodontics and stomatognathic physiology, equally contributed to diagnosing and writing. MK: specialist in infectious diseases, equally contributed to diagnosing, examination, and writing. JT: specialist in Clinical Neurophysiology, equally contributed to diagnosing, examination, and writing. MA: specialist in Neurology, equally contributed to diagnosing, examination, and writing.
